# Ultrafast charge generation in a homogenous polymer domain

**DOI:** 10.1038/s41598-022-13886-8

**Published:** 2022-06-16

**Authors:** Ruixuan Meng, Rui Zhu

**Affiliations:** School of Science, Shandong Jianzhu University, Jinan, 250100 Shandong Province China

**Keywords:** Condensed-matter physics, Semiconductors, Surfaces, interfaces and thin films

## Abstract

Efficient charge generation contributes greatly to the high performance of organic photovoltaic devices. The mechanism of charge separation induced by heterojunction has been widely accepted. However, how and why free charge carriers can generate in homogenous polymer domains remains to be explored. In this work, the extended tight-binding SSH model, combined with the non-adiabatic molecular dynamics simulation, is used to construct the model of a polymer array in an applied electric field and simulate the evolution of an excited state. It is found that under a very weak external electric field 5.0 × 10^−3^ V/Å, the excited state can evolve directly into spatially separated free charges at the femtosecond scale, and the efficiency is up to 97%. The stacking structure of the polymer array leads to intermolecular electron mutualization and forms intermolecular coupling. This interaction tends to delocalize the excited states in organic semiconductors, competing with the localization caused by electron–phonon coupling. Excitons within the homogenous polymer domains have lower binding energy, less energy dissipation, and ultrafast charge separation. Therefore, the initial excited state can evolve directly into free carriers under a very weak electric field. This finding provides a reasonable explanation for ultrafast charge generation in pure polymer phases and is consistent with the fact that delocalization always coexists with ultrafast charge generation. Moreover, the devices based on homogenous polymer domains are supposed to be stress-sensitive and performance-anisotropic since the above two interactions have contrary effects and work in perpendicular directions. This work is expected to bring inspiration for the design of organic functional materials and devices.

## Introduction

In research of organic photovoltaic (OPV) devices, power conversion efficiencies (PCE) are the primary concern^1–6^. The last five decades have seen its growing trend and the recent value has reached up to 19%^7^. Since the first-generation OPV, bulk heterojunction (BHJ) had become a necessary structure of active layers because the interfacial energy offset helps overcome exciton binding and realize the subsequent charge separation^7–13^. Ternary blend strategy and gap matching engineering are almost derived from this principle. However, this concept is challenged recently by the discovery of spontaneous charge generation in homogenous organic materials.

Several studies have shown pieces of evidence that charge generates in homogenous organic materials. In 2017, Veljko et al.^14^ attempted to find the reason why the space-separated charges appearing on 100 fs timescales were predominantly directly optically generated. This phenomenon was supported with their theoretical simulation to benefit from the ultrafast transition in a lattice model, rather than the interfaces of heterojunction. In Bakulin et al.^15^ experiment of 2020, the efficient charge generation was founded in the homojunction of α-sexithiophene. Although the charge generation is still due to the interface energy mismatch between the different phases, this is an important advance in the charge separation of homogenous materials. In early 2015, the direct measurement of long-lived charge-carrier generation was reported in chirality-pure, single-walled carbon nanotubes^16^. Despite the strong quantum confinement, as Park et al. stated, the unsymmetrical frontier orbital was suspected the primary cause. These suggest that BHJ is not necessary anymore for charge generation. Maxim et al.^17^ proved that efficient exciton-to-charge conversion could occur in star-shaped molecules films without an external acceptor. Besides in small molecular, the same phenomenon was also discovered in polymers. In the model semicrystalline system of Collins’ group study, decreasing the mixed-phase interface between pure polymer donor and pure acceptor domains has little effect on the efficiency of charge transfer (CT) state formation, but instead, dramatically increases the efficiency of CT state separation^18^. The fact is strongly illustrated that the heterojunction interface is not the only way to charge generation. In the Meager et al.^19^ comparison experiment, an obvious photocurrent enhancement was founded by moving the Alkyl-chain branching position away from the polymer backbone. They emphasized the stronger intermolecular $\pi - \pi$ stacking effect on photocurrent enhancement since the operation can improve the crystallinity of the polymer in the neat and blend films. The above studies collectively suggest a new pathway for charge generation beyond BHJ mechanism.

The question of charge generation in a homogenous organic semiconductor is of broad interest. Part of the explanations in the community is that defects are responsible for splitting excitons and creating charges since no synthesized organic material is defect-free. Nevertheless, some other studies place extra emphasis on the host material structure that delocalization in crystal suppress geminate recombination and boosts charge separation in organic solar cells^20^ and that intermolecular order is a prerequisite for free charge generation^21^. So far, there has been little agreement on the explicit mechanism. Meanwhile, larger homogenous domains can provide a continuous channel for charge transport, avoid phase segregation and improve device stability, which is a promising approach for optimizing device performance. Therefore, how and why the homogenous domains promote charge generation is a topic worthy of study and has practical significance.

This work aims to disclose the reason for spontaneous charge generation in homogenous polymer domains. A combination of quantitative calculation and qualitative analysis was used. By building the theoretical model of a conjugated polymer domain and performing the dynamical simulation of the initial excited state on it, the microscopic landscape of charge generation is shown. This work hopes to give a deeper understanding of spontaneous charge generation in terms of interactions. The remaining part of this paper is organized in the following way. Part two lays out the origin of the theoretical model and describes the meanings of parameters. The principle and advantages of the method are also mentioned in this part. Part three analyses the results and discusses the different aspects of spontaneous charge generation. The final part gives a summary and comment on the findings.

## Model and method

Based on the fact that most previous research only gives a simple relationship of material structures and photoinduced currents but lack direct observations, this work intends to present a microscopic landscape of charge generation by dynamic simulations. The extended tight-binding Su–Schrieffer–Heeger (SSH) model and the non-adiabatic kinetic method are employed. This method is particularly useful in studying low-dimensional organic conjugated systems for the advantage of emphasizing the electron–phonon (e–p) coupling. It also allows the visualization of microscopic dynamics.

**Figure 1:**
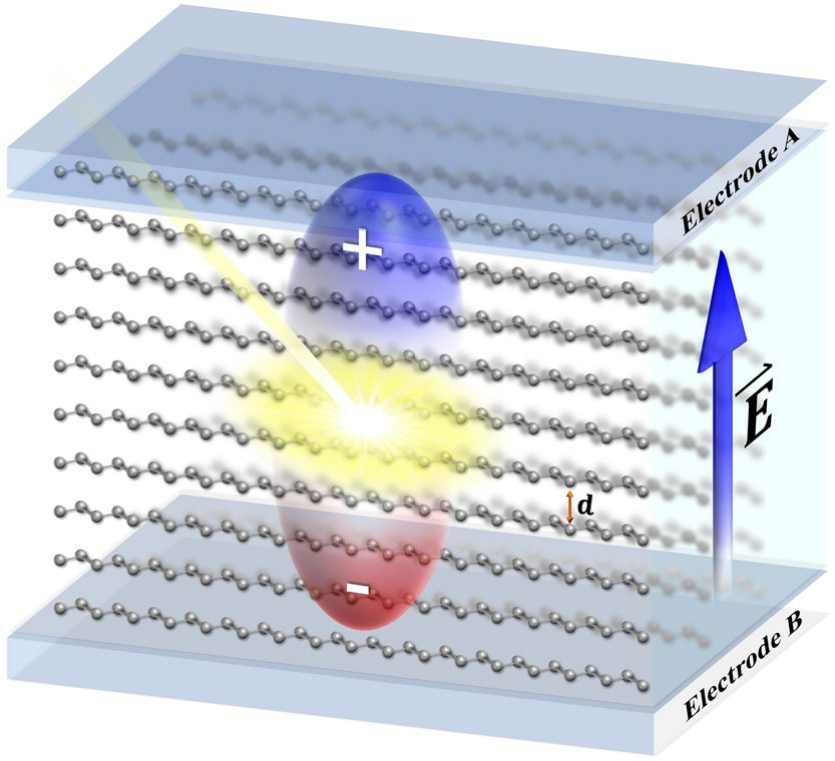
Schematic diagram of the polymer array model.

The intrinsic structure of the polymer domain and environmental factors are considered in the theoretical model. Conjugated polymers in homogenous domains are often arranged as a matrix. The itinerant $\pi$ electrons link neighbor chains together and construct interconnected domains. Since most of the conjugated polymer molecules have a planar structure, the intermolecular coupling is believed to construct in the $\pi - \pi$ stacking direction between the face-to-face adjacent molecules. While there is very less electron cloud overlap in the other vertical direction even though the molecules are adjacent to each other. Therefore, a polymer array in the stacking direction is built in the model. Sandwiched active layers are generally settled in electric fields that may come from the electrodes with different work functions, as well as trapped carriers, charged impurities, and interfacial energy offsets. Their strengths are roughly estimated at 1 ~ 100 × 10^−3^ V/Å^15,22^. The field orientations distribute randomly but can be decomposed along the polymer extended direction and perpendicular to this direction. It has been frequently proved that the component along the chain can help exciton intramolecular separation if the strength varies during 10^−2^ ~ 10^−1^ V/Å. Compared with it, the vertical component is more concerned in the current study for the dominant role in intermolecular charge generation. The simplified model was graphically depicted in Fig. 1. The Hamiltonian of the electron part is expressed as

1 $$ H = - \mathop \sum \limits_{m,n} t_{n,n + 1}^{m} \left( {C_{m,n + 1}^{ + } C_{m,n} + C_{m,n}^{ + } C_{m,n + 1} } \right) - \mathop \sum \limits_{m,n} t_{ \bot } \left( {C_{m,n}^{ + } C_{m + 1,n} + C_{m + 1,n}^{ + } C_{m,n} } \right) + \mathop \sum \limits_{m,n} \left| e \right|Emd\left( {C_{m,n}^{ + } C_{m,n} + C_{m,n}^{ + } C_{m,n} } \right) $$

The site $n \in \left( {1,100} \right)$ and chain index $m \in \left( {1,10} \right)$ construct a ten-polymer paralleled chain array. In the first term, $t_{n,n + 1}^{m} = t_{0} - \alpha \left( {u_{m,n + 1} - u_{m,n} } \right)$ stands for the transition between site $n$ and $n + 1$ on the $m$-th chain. It can be seen from the expression that electron behavior is coupled with lattice displacement $u_{m,n}$ via e–p coupling $\alpha$. The second term is the neighbor intermolecular correlation. $t_{ \bot }$ is influenced by intermolecular vertical distance $d$ in the form of $t_{ \bot } = t_{0} e^{{\left( {1 - \frac{d}{5}} \right)}}$^23^. The $d$ is allowed to vary from 1 to 10 Å to cover the practical conditions of highly crystalline phases of the polymer. In the last term, a uniform electric field potential was introduced of which the strength *E *= 5.0 × 10^−3^ V/Å is a constant.

Lattice behavior is treated classically with Newton’s function,

2 $$ M\ddot{u}_{m,n} = - K\left( {2u_{m,n} - u_{m,n + 1} - u_{m,n - 1} } \right) + 2\alpha \left( {\rho_{n,n + 1}^{m} - \rho_{n,n - 1}^{m} } \right) - \lambda M\dot{u}_{m,n} $$

On the right of the function, the first term refers that the neighbor lattice points are linked with spring oscillators, where *K* is the stiffness factor. The second term describes the influence of electron distribution on lattice motion. The density matrix is defined as $\rho_{{n,n^{^{\prime}} }}^{m} = \mathop \sum \limits_{\mu } \Psi_{\mu ,n}^{m} \left( t \right)f_{\mu } \Psi_{{\mu ,n^{^{\prime}} }}^{m*} \left( t \right)$. $f_{\mu }$ is a time-independent distribution function, which is set as 0, 1, or 2 depending on the initial occupation of the $\mu$-th level, and $n^{^{\prime}} = n \pm 1$. $\Psi_{\mu ,n}^{m} \left( t \right)$ is the electron wave function, and its evolution depends on the time-dependent Schrödinger equation

3 $$ i\hbar \frac{\partial }{\partial t}\Psi_{\mu ,n}^{m} \left( t \right) = H\Psi_{\mu ,n}^{m} \left( t \right) $$

In the last term of (2), a damping term $\lambda$ is introduced to describe the energy dissipation into the surrounding medium^24^.

The non-adiabatic dynamic process is expressed by the solution of the coupled derivative equations, which consist of the time-dependent Schrödinger Eq. (3) and Newton’s equation of motion (2). It can be solved by the Runge–Kutta method of order eight with step-size control, which has been widely used and proven to be an effective approach in the study of dynamic processes in conjugated polymers^25,26^. The basic parameters are set as $t_{0} = 2.5{\text{ eV}}$, *α *= 4.5 eV/Å, *K *= 21.0 eV/Å^2^, *M *= 1349.14 eV·fs^2^/Å^2^, which are determined as the standard data of polyacetylene via matching experimental gap and theoretical calculations^27^.

## Result and discussion

Free charge carriers are generally thought of as the product of Frenkel exciton, a localized quasi-particle in organic semiconductors. So, the necessary process after the light absorption consists of excited-state relaxation, transport, interfacial charge transfer, and final separation. Internal quantum efficiency is inevitably reduced during these steps because dissipation and recombination synchronously exist. Direct charge generation can realize a high efficiency via minimizing energy loss and increasing separation rate. This part presents the charge generation dynamics and reveals the underlying mechanism.

**Figure 2:**
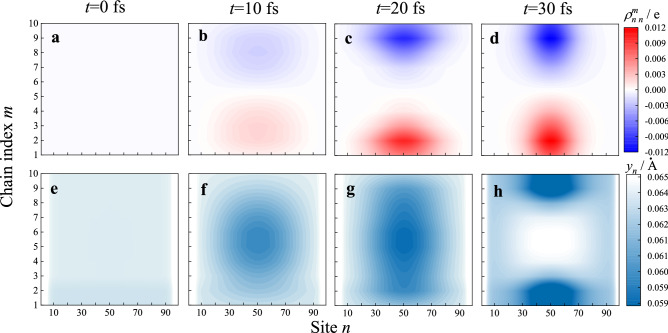
The evolution of charge density and lattice distortion in polymer array.

Dynamic simulations are set to start after photon absorption. The charge density distribution $\rho_{n,n}^{m}$ and smooth lattice configuration $y_{n} = \left( { - 1} \right)^{n} \left( {u_{n + 1} + u_{n - 1} - 2u_{n} } \right)/4$ (unit: Å) are recorded to trace dynamics. Intermolecular distance is set as *d *= 7 Å and the corresponding coupling strength is $t_{ \bot } = 0.17\;{\text{eV}}$. The representative states at the critical time nodes have been listed in Fig. 2. The system evolution begins with an excited state at $t = 0$, when one electron occupies the bottom of the conduction band but the lattice remains periodical. At this moment, there is no net charge distribution $\rho_{n,n}^{m} = 0$ and all the $y_{n}$ keep a constant. The system needs to experience relaxation for minimizing the total energy of the system. During the subsequent 20 fs, instead of forming a localized exciton, both the positive and negative charges synchronously generate and accumulate close to each edge. At the same time, the lattice distortion arises and expands on the central area of the array. The hetero-charges are spatially separated but trapped in a common lattice distortion, which is called polarized exciton or binding polaron pair. However, in the following short time, the lattice distortion evolves into two parts, and each is coupled with a certain amount of charge finally.

Four advantages are worthy of attention in the process. First, the weak field strength is enough for charge generation. The applied electric field is in the above process *E *= 5.0 × 10^−3^ V/Å, an order of magnitude smaller than that of intramolecular separation. The above condition is easy to satisfy in practical materials. Second, it indicates that internal quantum efficiency of over 90% is expected to be achieved in practical devices. Third, a very short time is needed for charge generation, which can be sorted to an ultrafast timescale. Forth, the “hot” or “cold” CT process is avoided, as well as the exciton transport, which means a direct charge separation. A similar case was proved in Chan’s study last year that spontaneous exciton dissociation would occur steadily and robustly in a small molecular lattice, although exciton dissociation into free charge carriers is an energy-uphill process^28^.

**Figure 3:**
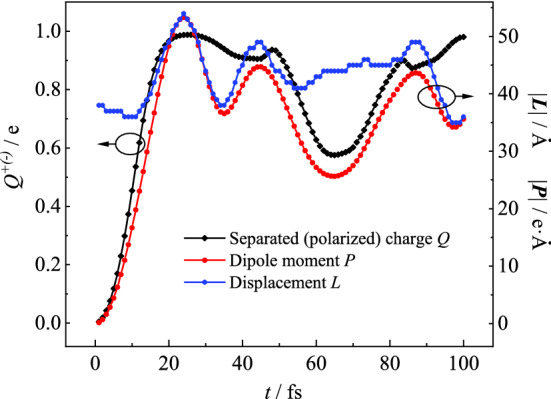
The displacement $\mathop{L}\limits^{\rightharpoonup}$, polar torque $\mathop{P}\limits^{\rightharpoonup}$ and quantity $Q^{ + \left( - \right)}$ of the spatial-separated charge are time-dependent in the charge generation process.

Since the time and ratio of charge generation are the important aspects of internal quantum efficiency, the related time-resolved quantities are shown in Fig. 2. The displacement $\mathop{L}\limits^{\rightharpoonup}$ and polar torque $\mathop{P}\limits^{\rightharpoonup}$ of electron–hole pair are defined to justify whether the exciton separates or not. $Q^{ + \left( - \right)} = \mathop \sum \limits_{m,n} \rho_{n,n}^{m}$ is the sum of positive (negative) charges, where $\rho_{n,n}^{m} \le \left( > \right)0$. $\mathop{L}\limits^{\rightharpoonup} = - \frac{{\sum \rho_{n,n}^{m} \mathop{r}\limits^{\rightharpoonup} _{m,n} }}{{\left| {Q^{ + \left( - \right)} } \right|}}$ is defined as the polarization displacement between the geometric centers of heterocharges. $\mathop{P}\limits^{\rightharpoonup} = - \sum \rho_{n,n}^{m} \mathop{r}\limits^{\rightharpoonup} _{m,n}$, varies with the distance and quality of the separated charge. In principle, once the charge separation finishes, $Q^{ + \left( - \right)}$ will reach its saturation. At the same time, the variations of $\mathop{P}\limits^{\rightharpoonup}$ and $\mathop{L}\limits^{\rightharpoonup}$ start to share the same pace. In Fig. 3, the quantity of separated (polarized) charge increases sharply from zero to approaching the unit during the initial 25 fs. The charge generation time $T_{cg}$ was identified 25 fs at present parameters, when the outcome of free charge reaches its first peak. After this time point, both the charge and lattice distortion are spatially separated and coupled to each other, in other words, free polarons have already formed, as shown in Fig. 2d, h. In the current model, since the moving polarons strike on the system boundary, rebound back and undergo some degree of charge recombination, the three quantities subsequently show some oscillations. During the above dynamic process, the charge generation efficiency $E_{cg} = \left| {Q_{\max }^{ + \left( - \right)} } \right| \times 100\%$ is identified as high as 97%. Ultrafast charge generation and high internal quantum efficiency are the two goals in the organic photovoltaic field. The excellent performance above should benefit from the stacking structure of the homogenous polymer domain.

**Figure 4:**
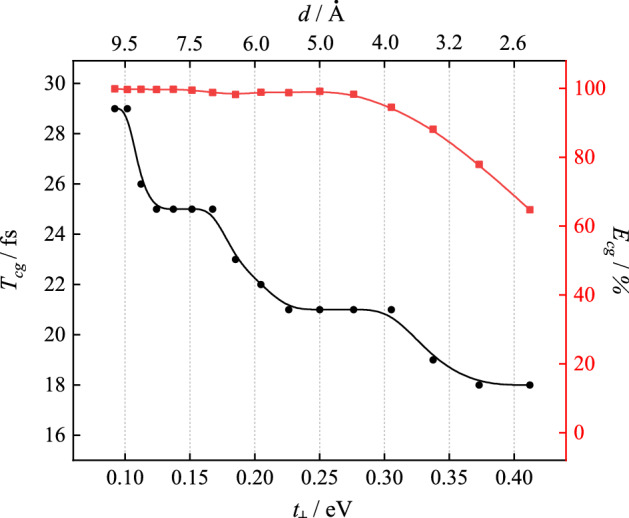
The dependence of charge generation time and efficiency on the intermolecular coupling (distance) in the organic molecular array.

The prominent difference between a homogenous polymer domain from an isolated chain is the intermolecular interaction which plays a key role in the charge generation. The variation of $E_{cg}$ and $T_{cg}$ on intermolecular coupling $t_{ \bot }$ (or intermolecular distance $d$) are calculated and summarized in Fig. 4. The $E_{cg}$ maintains a high level and only slightly drops in the strong coupling region. The $T_{cg}$ shows a continuous downward trend. It indicates that both the charge generation efficiency and generation speed can concurrently remain at a high level in a homogenous domain, in which the intermolecular coupling can vary in a relatively wide range.

A deeper understanding of the intrinsic nature of the homogenous domain is beneficial for material strategy and microstructure control. Steady-state calculations show some intrinsic properties of homogenous polymer domains in Table 1. The following points should be helpful to reveal the inherent mechanism of the ultrafast charge generation. First, for the ground state, the optical band gap narrows, and the bandwidth broadens with coupling intensity. It indicates that polymer arrays can broaden absorption speculum to some extent and make full use of solar energy. Second, a polymer array is a good solution for energy loss. The energy dissipation when the initial excited state directly relaxes into an energy-lowest exciton is calculated. Less thermal dissipation is found to happen in a more tightly coupled system. Third, Energy barriers between a relaxed exciton and free charge carriers are almost removed. Previous studies give pieces of evidence that BHJ energy offset needs to be large enough to overcome the exciton binding energy of 0.2–1.0 eV^29,30^. However, the tightly-stacking polymer array can make exciton binding energy down to 0.02 eV, a value comparable to thermal energy (0.026 eV). It ought to be one of the reasons why ultrafast charge separation has been frequently observed in most OPV systems. Forth, polymer arrays allow direct charge generation in terms of energy. The energy difference $E_{sep}$ is calculated if an initial excited-states do not relax but experience a direct charge separation. Data shows that the system energy of free carriers is lower than that of the initial excited state. Accordingly, the direct charge generation could be an exothermic process in polymer arrays. Fifth, the excited state owns a greater possibility to direct charge generation than a relaxed exciton in polymer arrays. The initial excited state has two choices, either separating to free charge carriers or relaxing to a localized exciton. According to the Franck–Condon principle that quantum transitions are much easier to occur at energy-adjacent states, the comparison of $E_{bin}$ and $E_{sup}$ shows the preference for direct charge generation.

**Table 1:** Parameters of organic stacking system with different intermolecular coupling strengths. (Unit: eV).

States	Ground state		Relaxed exciton		Energy dissipation of excited state relaxation	Exciton binding energy	Energy supply in firect charge generation
$t_{ \bot }$	Bandgap	Bandwidth	Bandgap	Bandwidth	*E*_*dis*_	*E*_*bin*_	*E*_*sup*_
0.00	2.456	9.968	0.912	10.011	0.660	0.430	−0.230
0.05	2.264	10.160	0.910	10.167	0.480	0.412	−0.068
0.10	2.072	10.352	0.906	10.358	0.325	0.308	−0.017
0.15	1.880	10.544	0.905	10.550	0.194	0.178	−0.016
0.20	1.688	10.736	1.560	10.741	0.041	0.025	−0.016
0.25	1.497	10.928	1.397	10.932	0.038	0.022	−0.016
0.30	1.305	11.119	1.213	11.124	0.037	0.021	−0.016
0.35	1.113	11.311	1.025	11.316	0.036	0.021	−0.016
0.40	0.921	11.503	0.836	11.507	0.036	0.020	−0.016

**Figure 5:**
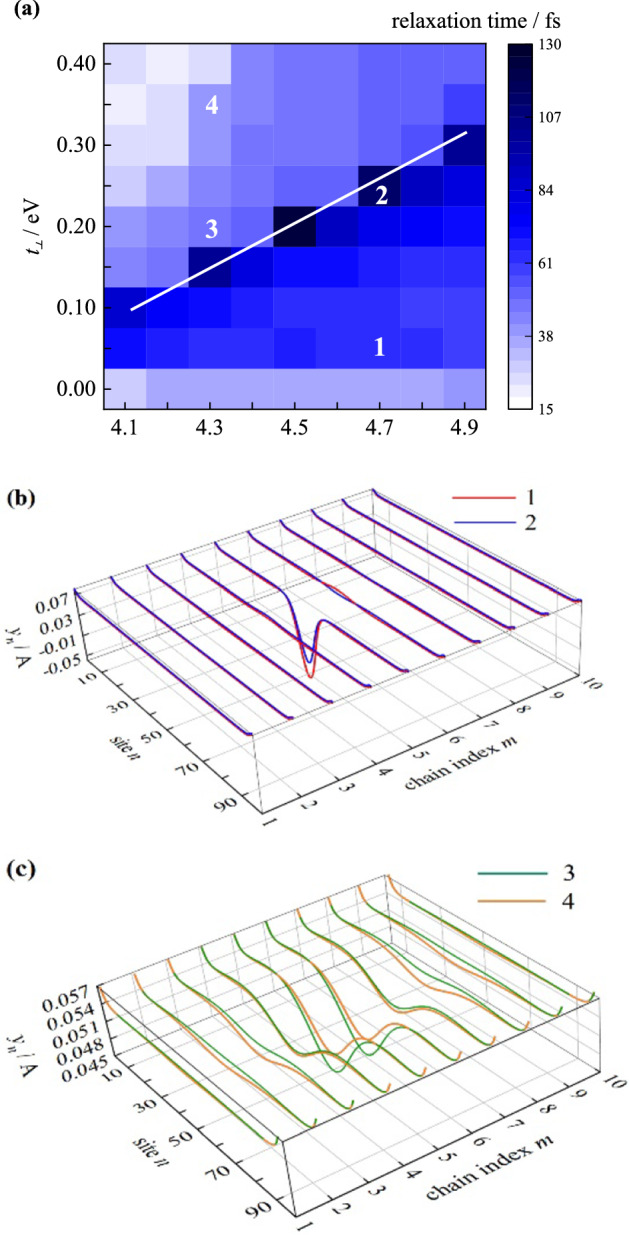
(**a**) The dependence of exciton relaxation time on intermolecular coupling and intramolecular e–p coupling; (**b**) and (**c**) the relaxed lattice displacement of sample points labeled in “a”.

Another remarkable feature of polymer arrays is that the quasi-particles are relatively delocalized, which might be responsible for ultrafast charge generation. It is widely acknowledged that the strong e–p coupling in organic semiconductors is the main reason for localization^31,32^. Once photoexcited or charge injected, the polymer backbone commonly sacrifices its periodic structure and forms a length of lattice distortion to minimize the total system energy. As a result, the physical processes in organic systems are often thought to proceed slowly^33,34^. Whereas the intermolecular coupling, which is only related to molecular distance, shows the opposite effect. The excited-state relaxation process is taken as an example for characterizing the competition effect between e–p coupling and intermolecular coupling. In Fig. 5a, the exciton relaxation time varies as the function of intramolecular “soft” e–p and intermolecular “hard” intermolecular coupling. The peak relaxation time appears at the intermediate region, signed with a straight line. In the bottom situations where the e–p coupling is dominant, the relaxation times are above 70 fs. As shown in Fig. 5b, both the excitons on sample points 1 and 2 are mainly located on an isolated chain, but the intermolecular coupling helps alleviate the intrachain lattice distortion. The slight difference in relaxation time is inferred to be mainly influenced by the degree of final lattice distortion. In the upper region of Fig. 5a, the exciton relaxation time was relatively short on the whole. From sample points 3 and 4, the relaxed exciton extends over several chains (Fig. 5c). At the same time, the relatively stronger intermolecular coupling results in a larger exciton coverage and a weaker lattice distortion. From the comparison of exciton size, the e–p coupling is mainly responsible for localization and the intermolecular coupling is the cause of delocalization. The cases in the strong intermolecular coupling region generally exhibit a faster relaxation process than those in the e–p coupling dominant region. This phenomenon suggests that a homogenous organic structure leads to a higher electron mutualization of frontier orbital, which constructs a better place for ultrafast charge generation. This finding is constant with the previous facts that ultrafast charge generation often coexists with delocalization^33^.

The above results are consistent with previous references that charge generation was frequently observed in the homogenous polymer domains^35^ and that delocalization often coexists with ultrafast charge separation^36–38^. Homogenous polymer domain leads to intermolecular electron mutualization and the competition between e–p coupling and intermolecular coupling regulates the degree of excited-state delocalization. These two facts contribute to low binding energy, less energy dissipation, and an ultrafast separation process. As a result, under a very weak electric field, can generate the free charge carriers. This mechanism is one of the reasonable explanations for ultrafast charge generation in homogenous polymer domains.

Several limitations in this study need to be acknowledged. More practical factors are not fully considered in the current study, such as chemical defects, and disordered thermal fluctuation. As a result, the charge generation efficiency should be somewhat overestimated. Moreover, high energy excited states and hot CT states are not considered in this article, which can affect the coupling intensity, exciton formation, and free charge carrier recombination and absorption. Even so, this regulation that two different types of interactions control the competition between localization and delocalization is qualitatively available for other organic semiconductors. Moreover, the devices consisting of homogenous polymer domains are supposed to be stress-sensitive and anisotropic because the above two interactions have contrary effects and work in perpendicular directions. Combining spin-related interactions with this studied mechanism would also help develop a more comprehensive understanding of novel organic phenomena, such as the excited-ferromagnetism or anisotropic electroluminescence. These will give inspiration to the development of organic spin devices.

## Conclusion

In response to the difficulty that the BHJ mechanism cannot explain the direct charge generation in pure polymer phases, this work proposed a new mechanism. The extended SSH model, combined with the non-adiabatic dynamical method, was adopted in the study. A model of polymer array in the electric field was constructed to simulate the homogenous polymer domain sandwiched in electrodes. The dynamic evolution of an initial excited state was calculated and found to directly evolve into spatially separated positive and negative charges in the stacking direction. This process simultaneously owns three advantages, weak electric field condition, very efficient charge generation, and ultrafast charge generation process. Specifically, the charge generation efficiency of 97% and charge generation time of 25 fs were achieved with a very weak electric field *E *= 5.0 × 10^−3^ V/Å. In the homogenous polymer domain, the intermolecular interactions enhance the degree of electron mutualization, in which the excited state can remain relatively delocalized, and own lower binding energy, less energy dissipation, and ultrafast charge generation. The excellent performance should benefit from the stacking structure of the polymer array and can be maintained over a wide intermolecular coupling range. This study also reveals the opposite effects that intramolecular e–p coupling is responsible for localization and the intermolecular coupling is the cause of delocalization. Because the two interactions act in perpendicular directions, and the intermolecular interactions are regulated by molecular spacing, the performance of polymer-based materials is supposed to be anisotropic and stress-sensitive. The findings are expected to bring inspiration for the design of organic functional devices.

## Data Availability

All data generated or analysed during this study are included in this published article.
